# Mutation of Influenza A Virus PA-X Decreases Pathogenicity in Chicken Embryos and Can Increase the Yield of Reassortant Candidate Vaccine Viruses

**DOI:** 10.1128/JVI.01551-18

**Published:** 2019-01-04

**Authors:** Saira Hussain, Matthew L. Turnbull, Helen M. Wise, Brett W. Jagger, Philippa M. Beard, Kristina Kovacikova, Jeffery K. Taubenberger, Lonneke Vervelde, Othmar G. Engelhardt, Paul Digard

**Affiliations:** a The Roslin Institute and Royal (Dick) School of Veterinary Studies, University of Edinburgh, Edinburgh, United Kingdom; b Department of Pathology, University of Cambridge, Cambridge, United Kingdom; c National Institutes of Health, Bethesda, Maryland, USA; d The Pirbright Institute, Pirbright, Surrey, United Kingdom; e National Institute for Biological Standards and Control, South Mimms, Hertfordshire, United Kingdom; St. Jude Children's Research Hospital

**Keywords:** PA-X, influenza, shutoff, vaccine

## Abstract

The PA-X protein of influenza A virus has roles in host cell shutoff and viral pathogenesis. While most strains are predicted to encode PA-X, strain-dependent variations in activity have been noted. We found that PA-X protein from the A/PR/8/34 (PR8) strain had significantly lower repressive activity against cellular gene expression than PA-X proteins from the avian strains A/turkey/England/50-92/91 (H5N1) (T/E) and A/chicken/Rostock/34 (H7N1). Loss of normal PA-X expression, either by mutation of the frameshift site or by truncating the X open reading frame (ORF), had little effect on the infectious virus titer of PR8 or PR8 7:1 reassortants with T/E segment 3 grown in embryonated hens’ eggs. However, in both virus backgrounds, mutation of PA-X led to decreased embryo mortality and lower overall pathology, effects that were more pronounced in the PR8 strain than in the T/E reassortant, despite the low shutoff activity of the PR8 PA-X. Purified PA-X mutant virus particles displayed an increased ratio of hemagglutinin (HA) to nucleoprotein (NP) and M1 compared to values for their wild-type (WT) counterparts, suggesting altered virion composition. When the PA-X gene was mutated in the background of poorly growing PR8 6:2 vaccine reassortant analogues containing the HA and neuraminidase (NA) segments from H1N1 2009 pandemic viruses or from an avian H7N3 strain, HA yield increased up to 2-fold. This suggests that the PR8 PA-X protein may harbor a function unrelated to host cell shutoff and that disruption of the PA-X gene has the potential to improve the HA yield of vaccine viruses.

**IMPORTANCE** Influenza A virus is a widespread pathogen that affects both humans and a variety of animal species, causing regular epidemics and sporadic pandemics, with major public health and economic consequences. A better understanding of virus biology is therefore important. The primary control measure is vaccination, which for humans mostly relies on antigens produced in eggs from PR8-based viruses bearing the glycoprotein genes of interest. However, not all reassortants replicate well enough to supply sufficient virus antigen for demand. The significance of our research lies in identifying that mutation of the PA-X gene in the PR8 strain of virus can improve antigen yield, potentially by decreasing the pathogenicity of the virus in embryonated eggs.

## Abstract (precis)

Influenza A virus is a widespread pathogen that affects both humans and a variety of animal species, causing regular epidemics and sporadic pandemics, with major public health and economic consequences. A better understanding of virus biology is therefore important. The primary control measure is vaccination, which for humans mostly relies on antigens produced in eggs from PR8-based viruses bearing the glycoprotein genes of interest. However, not all reassortants replicate well enough to supply sufficient virus antigen for demand. The significance of our research lies in identifying that mutation of the PA-X gene in the PR8 strain of virus can improve antigen yield, potentially by decreasing the pathogenicity of the virus in embryonated eggs.

## INTRODUCTION

Influenza epidemics occur most years as the viruses undergo antigenic drift. Influenza A viruses (IAV) and influenza B viruses cause seasonal human influenza, but IAV poses an additional risk of zoonotic infection, with the potential of a host switch and the generation of pandemic influenza virus. The 1918 Spanish flu pandemic was by far the worst, resulting in 40 to 100 million deaths worldwide (1), while the 2009 swine flu pandemic caused an estimated 200,000 deaths worldwide (2).

IAV contains eight genomic segments encoding at least 10 proteins. Six genomic segments (segments 1, 2, 3, 5, 7, and 8) encode the eight core internal proteins PB2, PB1, PA, nucleoprotein (NP), M1, NS1, and NS2, as well as the ion channel M2. These segments can also encode a variety of accessory proteins known to influence pathogenesis and virulence (reviewed in references 3 and 4). Segments 4 and 6 encode the two surface glycoproteins hemagglutinin (HA) and neuraminidase (NA), respectively (5, 6), and virus strains are divided into subtypes according to the antigenicity of these proteins.

Vaccination is the primary public health measure to reduce the impact of influenza epidemics and pandemics, principally using inactivated viruses chosen to antigenically match the currently circulating virus strains or newly emerging viruses of pandemic concern. However, before efficient vaccine production can commence, high-yielding candidate vaccine viruses (CVVs) need to be prepared. Seasonal CVVs are widely produced by classical reassortment. This process involves coinfecting embryonated hens’ eggs with the vaccine virus along with a high-yielding donor virus adapted to growth in eggs (most commonly the A/Puerto Rico/8/34 strain, or PR8). The highest-yielding viruses that contain the glycoproteins of the vaccine virus are then selected. Recombinant influenza viruses are also made by reverse genetics (RG) (7–9), which relies on the transfection of cells with plasmids engineered to express both viral genomic RNA and proteins from each of the eight segments and, hence, to initiate virus production; the resultant virus is subsequently amplified in eggs. When RG CVVs are made, typically the six segments encoding core proteins (backbone) are derived from the donor strain whereas the two segments encoding the antigens are derived from the vaccine virus. Classical reassortment has the advantage that it allows the fittest natural variant to be selected, but the process can be time-consuming. In the case of a pandemic, large quantities of vaccine must be made available quickly. Moreover, RG is the only viable method for production of CVVs for potentially pandemic highly pathogenic avian influenza viruses since it allows removal of genetic determinants of high pathogenicity in the virus genome as vaccines are manufactured in biosafety level 2 laboratories. A limited number of donor strains for IAV vaccine manufacture currently exist. Although PR8 is widely used, reassortant viruses based on it do not always grow sufficiently well for efficient vaccine manufacture. In the case of the 2009 H1N1 pandemic (pdm09), vaccine viruses grew poorly in eggs compared with growth of those for previous seasonal H1N1 isolates (10), resulting in manufacturers struggling to meet demand. Thus, there is a clear need for new reagents and methods for IAV production, particularly for a response to a pandemic.

**FIG 1:**
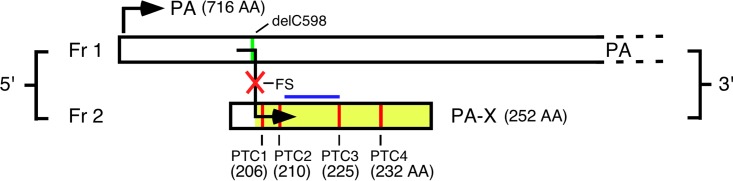
Mutational strategies used to alter IAV PA-X expression. The schematic diagram shows mutations in segment 3: a mutation at the frameshift (FS) site to generate a PA-X null virus, a mutation in the X ORF so that segment 3 expresses C-terminally truncated versions of PA-X (PTCs 1 to 4; size of products indicated), or removal of cytosine 598 (delC598) to place the X ORF in frame with PA such that only PA-X is expressed.

In recent years, several approaches have been employed to improve antigen yield of candidate vaccine viruses made by reverse genetics. These have involved empirical testing and selection of PR8 variants (11, 12), as well as targeted approaches such as making chimeric genes containing promoter and packaging signal regions of PR8 while encoding the ectodomain of the CVV glycoprotein genes (13–21) or introducing a wild-type (WT) virus-derived segment 2 (21–29). Our approach was to manipulate expression of an accessory protein virulence factor, PA-X (30). Segment 3, encoding PA as the primary gene product, also expresses PA-X by low-level ribosomal shifting into a +1 open reading frame (ORF), termed the X ORF (Fig. 1) (30). PA-X is a 29-kDa protein that contains the N-terminal endonuclease domain of PA and, in most isolates, a 61-amino-acid (aa) C terminus from the X ORF (30–32). It has roles in shutting off host cell protein synthesis and, at the whole-animal level, modulating the immune response (30, 33). Loss of PA-X expression has been shown to be associated with increased virulence in mice for 1918 H1N1, H5N1, and also pdm09 and classical swine influenza H1N1 strains, as well as in chickens and ducks infected with a highly pathogenic H5N1 virus (30, 34–40). However, in other circumstances, such as avian H9N2 viruses (40) or, in some cases, A(H1N1)pdm09 viruses (37, 41), mutation of PA-X resulted in reduced pathogenicity in mice. Similarly, a swine influenza H1N2 virus (42) lacking PA-X showed reduced pathogenicity in pigs. Moreover, PA-X activity in repressing cellular gene expression is strain dependent (33, 34, 40, 43), with laboratory-adapted viruses such as A/WSN/33 (WSN) showing lower levels of activity (33). Here, we show that although the PR8 PA-X polypeptide has low shutoff activity, removing its expression decreases the pathogenicity of the virus in the chicken embryo model. Moreover, we found that, for certain poorly growing CVV mimics, ablating PA-X expression improved HA yield from embryonated eggs up to 2-fold. In no case did loss of PA-X appear to be detrimental to the growth of CVVs, making it a potential candidate mutation for incorporation into the PR8 CVV donor backbone.

(This article was submitted to an online preprint archive [44].)

## RESULTS

### The PR8 virus strain PA-X has relatively low shutoff activity.

**FIG 2:**
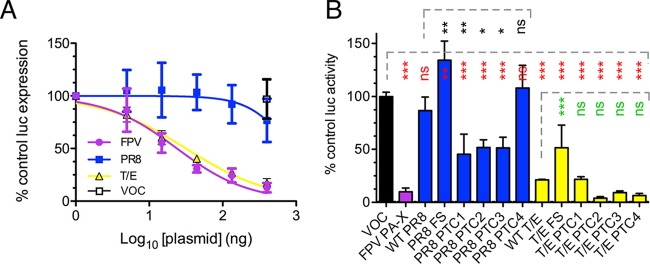
Virus strain-dependent variation in PA-X-mediated host cell shutoff activity. PA-X-mediated inhibition of cellular RNA polymerase II-driven gene expression in QT-35 cells. (A) Cells were cotransfected with 100 ng of pRL plasmid constitutively expressing *Renilla* luciferase and a dilution series of the indicated segment 3 pHW2000 plasmids or with a fixed amount of the empty pHW2000 vector (VOC). Luciferase (luc) activity was measured 48 h later and plotted as a percentage of the value for the pRL-only sample. Dose-inhibition curves were fitted using GraphPad Prism software. Data are means ± standard deviations of two independent experiments, each performed in triplicate. (B) Cells were cotransfected with 100 ng of pRL plasmid and 400 ng of effector pHW2000 plasmids expressing segment 3 products. Luciferase activity was measured 48 h later and plotted as a percentage of the value for a pHW2000 vector-only control. Data are the means ± standard deviations from two independent experiments performed in duplicate. Dashed lines indicate groups of statistical tests (against the left-hand bar in each case) as assessed by Dunnett's test (*, *P* < 0.05; **, *P* < 0.01; ***, *P* < 0.001; ns, not significant).

Previous work has noted variation in apparent activity of PA-X proteins from different strains of virus, with the laboratory-adapted strain WSN showing lower activity than many other strains (33). Reexamination of evidence concerning a postulated proteolytic activity of PA (43) suggested that lower PA-X activity might also be a feature of the PR8 strain. To test this, the ability of PR8 segment 3 gene products to inhibit cellular gene expression was compared to that of two avian virus-derived PA segments (from A/chicken/Rostock/34 [H7N1; FPV] and A/turkey/England/50-92/91 [H5N1; T/E]. Avian QT-35 (Japanese quail fibrosarcoma) cells were cotransfected with a consistent amount of a plasmid encoding luciferase under the control of a constitutive RNA polymerase II promoter (pRL) and increasing amounts of the IAV cDNAs (in pHW2000-based RG plasmids) or, as a negative control, with the maximum amount of the empty pHW2000 vector. Luciferase expression was measured 48 h later and expressed as a percentage of the amount obtained from pRL-only transfections. Transfection of a 4-fold excess of empty pHW2000 vector over the luciferase reporter plasmid had no significant effect on luciferase expression, whereas cotransfection of the same amount of either the FPV or T/E segments suppressed activity to around 10% of the control level (Fig. 2A). Titration of the FPV and T/E plasmids gave a clear dose-response relationship, giving estimated 50% effective concentration (EC_50_) values of 24 ± 1.1 ng and 32 ± 1.1 ng, respectively. In contrast, the maximum amount of the PR8 plasmid inhibited luciferase expression by only around 30%, and an EC_50_ value could not be calculated, indicating a lower ability to repress cellular gene expression. Similarly, low inhibitory activity of the PR8 segment 3 was seen in a variety of other mammalian cell lines (data not shown), suggesting that it was an intrinsic feature of the viral gene rather than a host- or cell-type-specific outcome.

Several studies have shown the X ORF to be important in the host cell shutoff function and virulence of PA-X (37, 45–47). To further explore the influence of X ORF sequences on virus strain-specific host cell shutoff, mutations were constructed in segment 3 in which PA-X expression was either hindered (via mutation of the frameshift [FS] site) or altered by the insertion of premature termination codons (PTC1 to PTC4; silent in the PA ORF) such that C-terminally truncated forms of PA-X would be expressed (Fig. 1). QT-35 cells were cotransfected with the pRL plasmid and a fixed amount of WT, FS, or PTC plasmid, and luciferase expression was measured 48 h later. As before, the WT FPV and T/E PA-X proteins reduced luciferase activity by approximately 5- to 10-fold, while WT PR8 PA-X had no significant effect (Fig. 2B). Introducing the FS mutation into both the PR8 and T/E segment 3s significantly increased luciferase activity relative to that of the WT construct. Truncation of the PR8 PA-X to 225 aa or less (PTC mutations 1 to 3) significantly improved shutoff activity although not to the levels seen with the WT avian virus polypeptides, while the PTC4 truncation had no effect. In contrast, none of the PTC mutations significantly affected activity of the T/E PA-X although there was a trend toward increased activity from the PTC2, PTC3, and PTC4 truncations.

**FIG 3:**
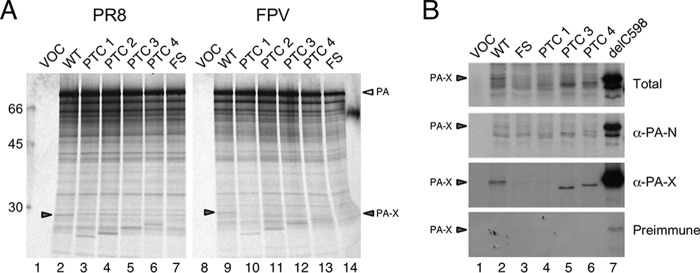
Expression of PA-X polypeptides *in vitro*. Aliquots of rabbit reticulocyte lysate supplemented with [^35^S]methionine were programmed with WT or mutated (as indicated) segment 3 plasmids or with empty plasmid vector (VOC), and the resulting radiolabeled polypeptides were visualized by SDS-PAGE and autoradiography before (A) or after (B) immunoprecipitation with the indicated antisera. Arrowheads in panel A indicate full-length PA-X while molecular mass (kDa) markers are shown on the left.

Low activity could be due to decreased expression and/or decreased activity of PA-X. To examine this, expression levels of the low-activity PR8 and high-activity FPV PA-X constructs were compared by *in vitro* translation (IVT) reactions in rabbit reticulocyte lysate. Translation of segment 3s from both PR8 and FPV produced both full-length PA and similar quantities of a minor polypeptide species of the expected size for PA-X, whose abundance decreased after addition of the FS mutation or whose electrophoretic mobility was altered in stepwise fashion after C-terminal truncation with the mutations of PTC1 to PTC4 (Fig. 3A). This suggested that differences in shutoff potential were not linked to intrinsic differences in PA-X protein synthesis. To confirm the identity of the PR8 *in vitro*-translated polypeptides, immunoprecipitations of IVT products with serum raised against either the N-terminal domain of PA or an X ORF-derived polypeptide or preimmune sera (30) were performed (Fig. 3B). WT PA-X was clearly visible in samples immunoprecipitated with anti-PA-X and anti-PA-N but not with the preimmune serum, where it comigrated with the product from the delC598 plasmid, a construct in which cytosine 598 of segment 3 (the nucleotide skipped during the PA-X frameshifting event [48]) had been deleted to put the X ORF into the same reading frame as the N-terminal PA domain (Fig. 3B, lanes 2 and 7). In contrast, only background amounts of protein were precipitated from the FS IVT (Fig. 3B, lane 3). Faster-migrating polypeptide products from the PTC3 and PTC4 plasmids showed similar reactivities to WT PA-X (Fig. 3B, lanes 5 and 6) whereas the product of the PTC1 plasmid was precipitated only by anti-PA-N (lane 4), as expected because of the loss of the epitope used to raise the PA-X antiserum (Fig. 1). Overall, therefore, the PR8 PA-X polypeptide possessed lower shutoff activity than two avian virus PA-X polypeptides despite comparable expression levels *in vitro*, and its activity could be modulated by mutation of the X ORF.

### Loss of PA-X expression results in significantly less pathogenicity in chick embryos without affecting virus replication.

**FIG 4:**
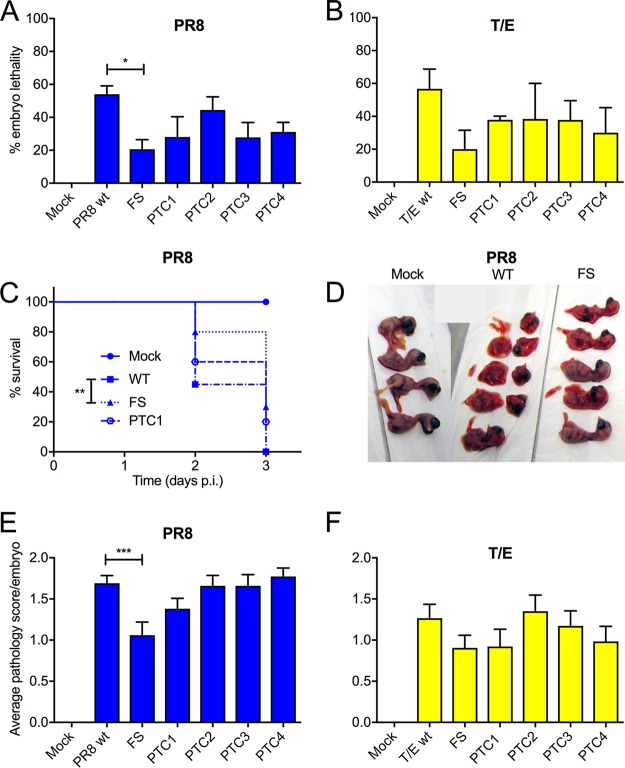
Effect of PA-X mutations in a chicken embryo pathogenicity model. Groups of 5 to 6 embryonated hens’ eggs were infected with 1,000 PFU of the indicated viruses, and embryo viability was determined by candling at 2 days p. i. (A and B). Data are plotted as means ± standard errors of the means of the percent embryo lethality from 3 to 4 independent experiments. Horizontal bars indicate statistical significance (*, *P* < 0.05) as assessed by Dunnett’s test. (C) Infected eggs were monitored daily for embryo viability, and survival was plotted versus time. Data are from three independent experiments with 5 to 10 eggs per experiment. Statistical significance between WT and FS viruses (**, *P* < 0.01) was assessed by a log rank (Mantel-Cox) test. (D to F) From the experiments described in panels A and B, embryos were imaged and scored blind by two observers as follows: 0, normal; 1, intact but bloody; 2, small, damaged, and with severe hemorrhages. (E and F) Data are the averages ± standard error of the means of the pathology scores from 3 to 4 independent experiments. The horizontal bar indicates statistical significance (***, *P* < 0.001) as assessed by Dunnett’s test.

In order to further characterize the role of PA-X as a virulence determinant, we tested the panel of high- and low-activity mutants in the chicken embryo pathogenicity model. Embryonated hens’ eggs were infected with PR8-based viruses containing either PR8 or T/E WT or mutant segment 3s, and embryo viability was monitored at 2 days postinfection (p.i.) by candling. Both WT PR8 and the WT 7:1 reassortant with the T/E segment 3 viruses had killed over 50% of the embryos by this point (Fig. 4A and B). Truncation of PA-X by the PTC mutations led to small improvements in embryo survival although none of the differences were statistically significant. However, embryo lethality was significantly reduced to below 20% following infection with the PR8 FS virus compared to the lethality of PR8 WT virus. A similar reduction in lethality was seen for the T/E FS virus although the difference was not statistically significant. This reduction in embryo pathogenicity following ablation of PA-X expression suggested potential utility as a targeted mutation in the PR8 backbone used to make CVVs. Accordingly, to characterize the effects of mutating PR8 PA-X over the period used for vaccine manufacture, embryo survival was monitored daily for 72 h. Eggs infected with WT PR8 showed 45% embryo survival at 2 days p.i., and all embryos were dead by day 3 (Fig. 4C). However, the PR8 FS-infected eggs showed a statistically significant improvement in survival compared to that of the WT, with 80% and 30% survival at days 2 and 3, respectively. Embryos infected with PR8 expressing the C-terminally truncated PTC1 form of PA-X showed an intermediate survival phenotype, with 60% and 20% survival at days 2 and 3, respectively.

To further assess the effects of mutating PA-X, the chicken embryos were examined for gross pathology. WT PR8 infection resulted in smaller, more fragile embryos with diffuse reddening, interpreted as hemorrhages (Fig. 4D). In comparison, the PA-X null FS mutant-infected embryos remained intact and were visibly larger and less red. To quantitate these observations, embryos were scored blind for gross pathology. Taking uninfected embryos as a baseline, it was clear that WT PR8 virus as well as the PA-X truncation mutants induced severe changes to the embryos (Fig. 4E). In contrast, the PA-X null FS mutant caused significantly less pathology. The WT 7:1 T/E reassortant virus gave less overt pathology than WT PR8, but, again, reducing PA-X expression through the FS mutation further reduced damage to the embryos (Fig. 4F). Similar trends in pathology were also seen with 7:1 PR8 reassortant viruses containing either WT or FS mutant versions of FPV segment 3 (data not shown).

**FIG 5:**
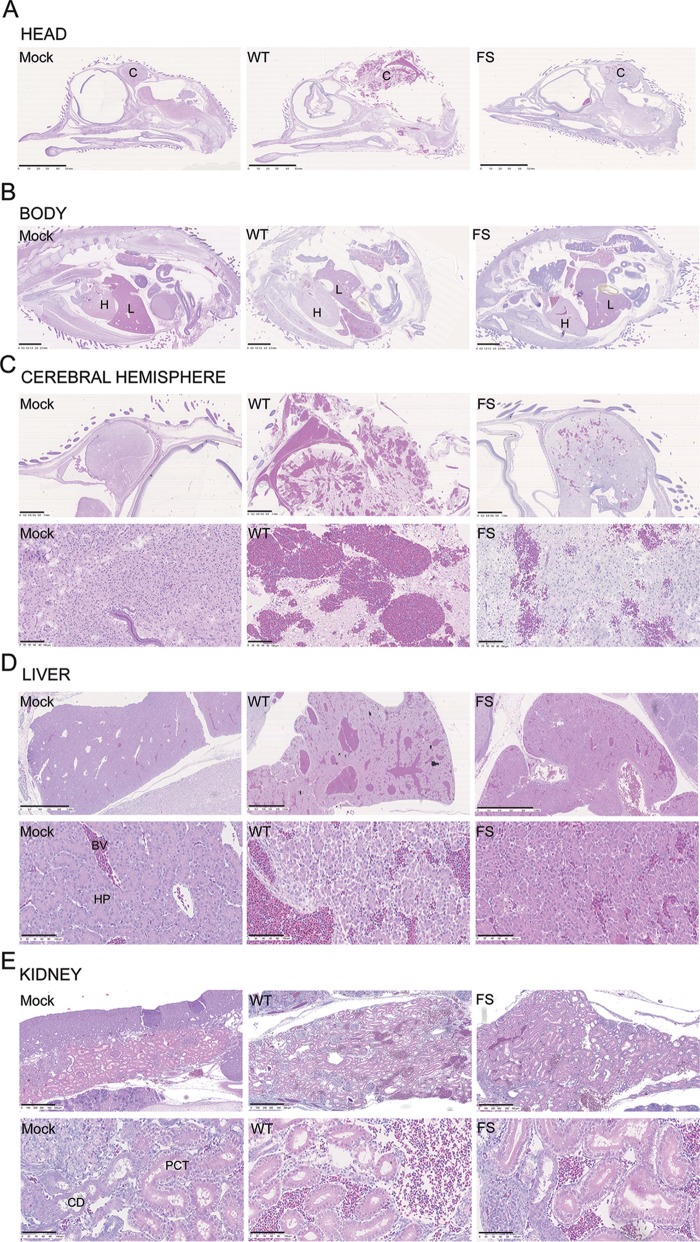
Histopathology of chick embryos following infection with PR8 viruses. Embryonated hens’ eggs were infected with segment 3 WT or mutant viruses or mock infected. At 2 days p.i. embryos were fixed, sectioned, and stained with H&E before imaging with a NanoZoomer XR instrument (Hamamatsu) using bright-field settings. Representative pictures are shown as indicated. Scale bars: 5 mm (A), 2.5 mm (B), 1 mm (C and D, upper row), 500 μm (E, upper row) 100 μm (C to E, lower row). C, cerebral hemisphere; H, heart; L, liver; PCT, proximal convoluted tubule; CD, collecting duct; HP, hepatocytes; BV, blood vessel.

Examination of hematoxylin and eosin (H&E)-stained sections through the embryos revealed pathology in numerous organs, including the brain, liver, and kidney (Fig. 5). In the brain of embryos infected with WT virus, there was marked rarefaction of the neuropil, few neurons were identifiable, and there was accumulation of red blood cells (Fig. 5C). In the liver of embryos infected with WT virus, the hepatic cords were disorganized, and the hepatocytes were often separated by large pools of red blood cells (Fig. 5D). In the kidney of embryos infected with WT virus, tubules were often lined by degenerate epithelial cells (characterized by loss of cellular detail) (Fig. 5E). In all cases, the pathology noted in WT virus-infected embryos was also present in the FS virus-infected embryos but with reduced severity. Thus, overall, disruption of PA-X expression in PR8 resulted in significantly less pathogenicity in chick embryos.

**FIG 6:**
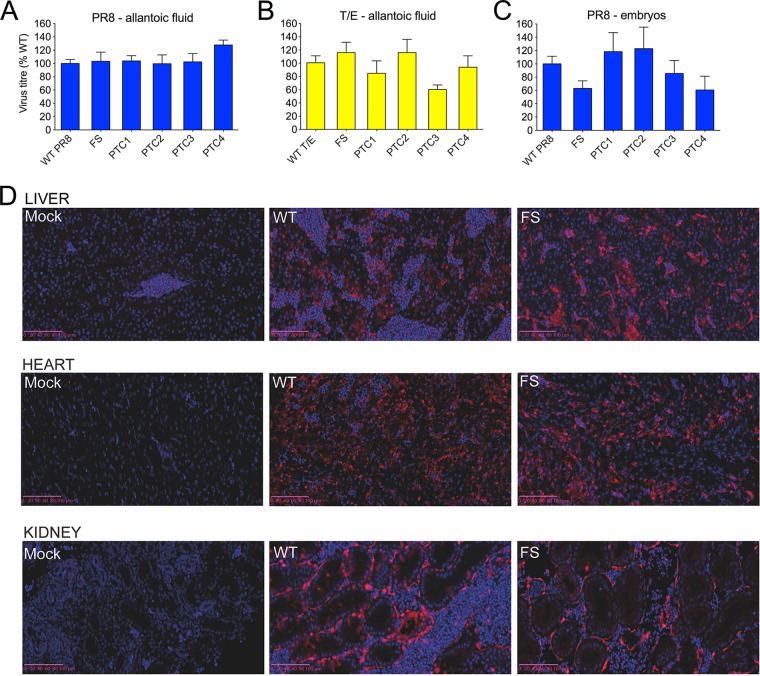
Effects of mutating PA-X expression on virus replication in chick embryos. Groups of 5 to 6 embryonated hens’ eggs were infected with the indicated viruses, and at 2 days p.i., virus titers were determined by plaque assay from allantoic fluid (A and B) or from washed and macerated chick embryos (C). Graphs represent the means ± standard errors of the mean s from three (A and B) or two to four (C) independent experiments. Titers of mutant viruses were not significantly different from the WT virus titer (Dunnett’s test). (D) Embryos were fixed at 2 days p.i., sectioned, and stained for IAV NP and DNA before imaging was performed using a NanoZoomer XR instrument (Hamamatsu) on fluorescence settings. Representative images of liver, heart, and kidney are shown. Scale bar, 100 μm. Red, NP; blue, DAPI.

Reduced pathogenicity *in vivo* following loss of PA-X expression could be due to a replication deficiency of the virus although the viruses replicated equivalently in mammalian MDCK cells (data not shown). To test if replication did differ *in ovo*, infectious virus titers were obtained (by plaque titration on MDCK cells) from the allantoic fluid of embryonated hens’ eggs infected with the panels of PR8 and T/E viruses at 2 days p.i. However, there were no significant differences in titers between either PR8 or T/E WT and PA-X mutant viruses (Fig. 6A and B). Since the reduced pathogenicity phenotype *in ovo* on loss of PA-X expression was more pronounced for viruses with PR8 segment 3 than for those with the T/E gene, embryos from PR8 WT and segment 3 mutant-infected eggs were harvested at 2 days p.i., washed, and macerated, and virus titers from the homogenates were determined. Titers from embryos infected with the PR8 FS and PTC4 viruses were slightly (less than 2-fold) reduced compared to titers of embryos infected with PR8 WT virus (Fig. 6C), but overall there were no significant differences in titers between the viruses. To see if there were differences in virus localization in tissues between PR8 WT and FS viruses, immunohistochemistry was performed on chicken embryo sections to detect viral NP as a marker of infected cells. NP-positive cells were seen in blood vessels throughout the head and body of both PR8 WT- and FS-infected embryos; liver, heart and kidney are shown as representatives (Fig. 6D), indicating that the circulatory system had been infected. However, there were no clear differences in virus localization between embryos infected with the WT and those infected with FS viruses.

Overall, therefore, the loss of PA-X expression reduced IAV pathogenicity in chick embryos, as assessed by mortality curves and both gross and histopathological examination of embryo bodies. This reduced pathogenicity did not appear to correlate with reduced replication or with altered distribution of the virus *in ovo*.

### Ablating PA-X expression alters virion composition.

**FIG 7:**
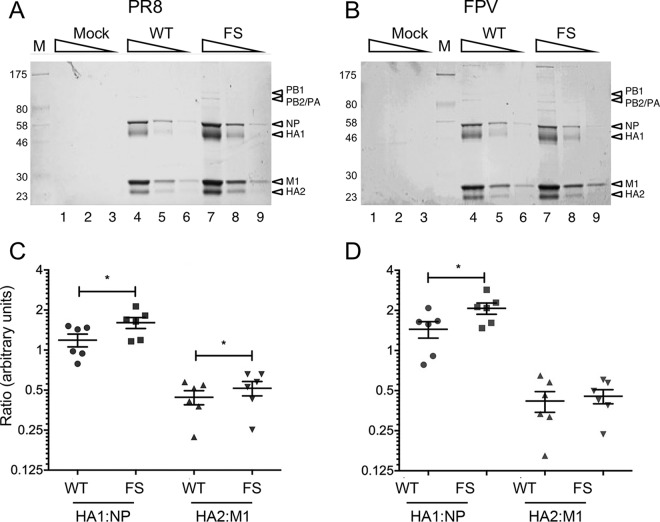
Virion composition of WT or FS mutant viruses. Embryonated hens’ eggs were infected with WT or segment 3 mutant viruses or mock infected. (A and B) At 2 days p.i., virus was purified from allantoic fluid by sucrose density gradient ultracentrifugation, and 3-fold serial dilutions were analyzed by SDS-PAGE on 10% polyacrylamide gels and staining with Coomassie blue. (C and D) For PR8 and FPV, respectively, the ratios of HA1/NP and HA2/M1 were determined by densitometry of SDS-PAGE gels. Scatter plots with the means and standard errors of the means of six measurements from three independent experiments using two independently rescued virus stocks are shown. Horizontal bars indicate statistical significance (*, *P* < 0.05) as assessed by paired *t* test.

Other viruses encode host control proteins with mRNA endonuclease activity, including the SOX protein of murine gammaherpesvirus MHV68, whose expression has been shown also to modulate virion composition (49). Also, egg-grown IAV titer and HA yield do not always exactly match, with certain problematic CVVs containing smaller amounts of HA per virion (16, 50, 51). Accordingly, we compared the relative quantities of virion structural proteins between PA-X-expressing and PA-X null viruses. Two pairs of viruses were tested: either an entirely PR8-based virus or a 7:1 reassortant of PR8 with FPV segment 3, both with and without the FS mutation. Viruses were grown in eggs as before and purified from allantoic fluid by density gradient ultracentrifugation before polypeptides were separated by SDS-PAGE and visualized by staining with Coomassie blue. To ensure that overall differences in protein loading did not bias the results, 3-fold dilutions of the samples were analyzed. From the gels, the major virion components of both WT and FS virus preparations could be distinguished: NP, the two cleaved forms of hemagglutinin, HA1 and HA2, the matrix protein, M1, and in lower abundance, the polymerase proteins (Fig. 7A and B, lanes 4 to 9). In contrast, only trace polypeptides were present in similarly purified samples from uninfected allantoic fluid (Fig. 7A and B, lanes 1 to 3). Densitometry was used to assess the relative viral protein contents of the viruses. The two most heavily loaded lanes (where band intensities were sufficient for accurate measurement) were quantified, and average HA1/NP and HA2/M1 ratios were calculated. When the data from three independent experiments were examined in aggregate by scatter plot, a statistically significant increase in the average quantity of HA1 relative to that of NP was evident for both PR8 and the FPV reassortant FS viruses of ∼1.4-fold and ∼1.6-fold, respectively, compared to the level of the WT (Fig. 7C and D). The ratio of HA2/M1 was also significantly increased in the PR8 FS virus (∼1.2- fold greater than that of the WT), and a similar but nonsignificant increase was seen for the FPV virus pair. These data are consistent with the hypothesis that PA-X expression modulates virion composition.

### Ablating PA-X expression increases HA yield of CVVs bearing pdm09 glycoproteins.

**FIG 8:**
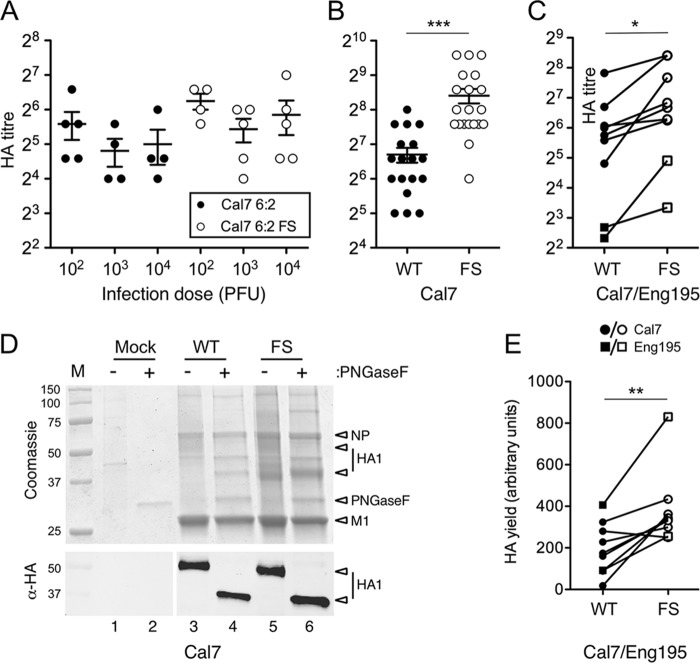
Effect of the PA-X FS mutation on HA yield of A(H1N1)pdm09 CVV mimics. (A to C) Embryonated hens’ eggs were infected as indicated, and HA titers in allantoic fluid were measured at 3 days p.i. for groups of 5 (A) or 20 eggs (B) per condition. Scatter plots of titers from individual eggs with means and standard errors of the means are shown. (***, *P* < 0.001; assessed by unpaired *t* test). Average HA titers from groups of eggs inoculated at the infection dose which gave maximum yield are shown as paired observations (C). Statistical significance was assessed by a paired *t* test (*, *P* < 0.05; *n* = 9). (D and E) Allantoic fluid was clarified, and virus was pelleted by ultracentrifugation through 30% sucrose pads. Equal volumes of resuspended virus pellets were separated by SDS-PAGE on a 12% polyacrylamide gel and visualized by staining with Coomassie blue (upper panel) or Western blotting for HA1 (lower panel) with (+) or without (−) prior treatment with PNGase F (D). Molecular mass (kDa) markers and specific polypeptides are labeled. Deglycosylated HA1 yield was quantified by densitometry of the Western blots (E). Data points represent eight independent experiments using three independently rescued RG virus stocks shown as paired observations (**, *P* < 0.01, *n* = 8; as assessed by paired *t* test). Circles represent Cal7, and squares represent Eng195 CVV mimics.

The reduced pathogenicity and corresponding longer embryo survival time induced by the PR8 FS mutant *in ovo*, coupled with evident modulation of virion composition in favor of HA content, suggested a strategy to increase overall antigen yields for PR8-based CVVs. Therefore, the effect of incorporating the PA-X FS mutation into CVV mimics containing glycoproteins of different IAV subtypes was examined. Reasoning that a benefit might be most apparent for a poor-yielding strain, 6:2 CVV mimics containing the glycoprotein genes from the A(H1N1)pdm09 vaccine strain, A/California/07/2009 (Cal7), with the six internal genes from PR8, with or without the FS mutation in segment 3, were generated. Growth of these viruses in embryonated hens’ eggs was then assessed by inoculating eggs with either 100, 1,000, or 10,000 PFU per egg (modeling the empirical approach used in vaccine manufacture to find the optimal inoculation dose) and measuring the HA titer at 3 days p.i. Both viruses grew best at an inoculation dose of 100 PFU/egg, but final yield was both relatively low (as expected, ∼64 hemagglutinating units [HAU]/50 μl) and insensitive to input dose, with average titers varying less than 2-fold across the 100-fold range of inocula (Fig. 8A). However, at each dose, the 6:2 FS virus gave a higher titer (on average, 1.6-fold) than the parental 6:2 reassortant. In order to assess HA yield between the WT and FS viruses on a larger scale, comparable to that used by WHO Essential Regulatory Laboratories (ERLs) such as the National Institute for Biological Standards and Control (NIBSC), United Kingdom, 20 eggs per virus were infected at a single inoculation dose. In this experiment, the average HA titer of the FS virus was over 3 times higher than that of the WT 6:2 virus (Fig. 8B). To further determine the consistency of these results, HA titer yields were assessed from two independently rescued reverse genetics stocks of the Cal7 6:2 CVV mimics, with or without the PR8 PA-X gene, as well as from another 6:2 CVV mimic bearing the glycoproteins from the A/England/195/2009 (Eng195) A(H1N1)pdm09 strain. HA yield from different stocks was assessed in independent repeats of both small-scale (5 eggs for each of three different inoculation doses, taking data from the dose that gave maximum yield) and large-scale (20 eggs per single dose of virus) experiments. Examination of the average HA titers showed considerable variation between results of independent experiments (Fig. 8C). However, when titers were plotted as paired data points, it was obvious that in every experiment, the FS viruses gave a higher yield than the parental 6:2 reassortant, and, on average, there were 2.7- and 3.8-fold higher HA titers with the segment 3 FS mutation for Cal7 and Eng195, respectively (Table 1).

**TABLE 1:** Effects of the ΔPA-X FS mutation on HA yield of CVVs grown in eggs

Lineage and subtype	Strain	No. of independent rescues	HA titer^*a*^		Relative HA titer^*b*^	Relative HA1 yield^*b*^	No. of expts.		
			6:2 virus	6:2 FS virus			Total	Small scale	Large scale
Human pdm09									
H1N1	A/California/07/2009	2	106 ± 86.6	249 ± 109	2.65 ± 2.16	1.90 ± 1.07^*c*^	7	4	3
	A/California/07/2009 chimeric HA (NIBRG-119)	1				1.54 ± 0.430	2	1	1
	A/England/195/2009	1	5.71 ± 0.710	20.1 ± 9.92	3.79 ± 2.21	2.40 ± 0.370	2	1	1
Human pdm1968									
H3N2	A/Udorn/307/72	2	2200 ± 929	2100 ± 720	1.26 ± 0.470	1.35 ± 0.360	5	3	2
	A/Hong Kong/1/68	1	801 ± 117	843 ± 140	1.05 ± 0.06	1.22 ± 0.390	3	2	1
Avian									
H7N3	A/mallard/Netherlands/12/2000 (NIBRG-60)	2	33.5 ± 29.1	45.0 ± 36.9	1.34 ± 0.180	1.55 ± 0.140	5	3	2
H5N1	A/turkey/Turkey/1/2005/1/2005 (NIBRG-23)	2	47.1 ± 27.3	48.8 ± 23.2	1.13 ± 0.230	1.10 ± 0.300	5	3	2
H1N1	A/mallard/Netherlands/10/99	2	123 ± 59.0	128 ± 37.0	1.22 ± 0.370	1.13 ± 0.360	5	4	1
H9N2	A/chicken/Pakistan/UDL-01/2008	2	302 ± 364	312 ± 264	0.920 ± 0.300	1.01 ± 0.180	4	2	2

a Average HA titers of eggs incubated at 35°C. Values are means ± standard deviations.

b Ratio of the average HA titer or HA1 yield of FS (Δ PA-X) viruses to values for their WT counterparts (from eggs incubated at 35°C or 37.5°C). Values are means ± standard deviations.

c An outlier from one experiment was ignored in calculation of the average.

To directly assess HA protein yield, viruses were partially purified by ultracentrifugation of pooled allantoic fluid through 30% sucrose cushions. Protein content was analyzed by SDS-PAGE and Coomassie staining, either before or after treatment with peptide-*N*-glycosidase F (PNGase F) to remove glycosylation from HA and NA. Both virus preparations gave polypeptide profiles that were clearly different from those of uninfected allantoic fluid processed in parallel, with obvious NP and M1 staining, as well from other polypeptide species of less certain origin (Fig. 8D). Overall protein recovery was higher in the FS virus than in the WT reassortant virus (Fig. 8D, compare lanes 3 and 4 with lanes 5 and 6), but the poor yields of these viruses made unambiguous identification of the HA polypeptide difficult. However, PNGase F treatment led to the appearance of a defined protein band migrating at around 40 kDa that probably represented deglycosylated HA1, and this was present in appreciably higher quantities in the 6:2 FS preparation (Fig. 8D, compare lanes 4 and 6). Therefore, equivalent amounts of glycosylated or deglycosylated samples from the Cal7 WT and FS reassortants were analyzed by SDS-PAGE and Western blotting using anti-pdm09 HA serum. The Western blot gave a clear readout for HA1 content, confirmed the mobility shift upon deglycosylation, and showed increased amounts of HA1 in the 6:2 FS samples (Fig. 8D, lower panel). Quantitative measurements of the deglycosylated samples showed that the 6:2 FS virus gave a 1.9-fold-greater HA1 yield than the WT reassortant. To test the reproducibility of this finding, HA1 yield was assessed by densitometry of deglycosylated HA1 following SDS-PAGE and Western blotting for partially purified virus from nine independent experiments with the Cal7 and Eng195 reassortants. When results were examined as paired observations, it was evident that in 8 of the 9 experiments, the FS viruses gave greater HA yields than the parental virus, with only one experiment producing a smaller amount (Fig. 8E). In one large-scale experiment, the HA1 yield of 6:2 FS was approximately 20-fold higher than that of its 6:2 counterpart. However, in all other experiments, the 6:2 FS virus gave between 1.5- and 3-fold increases in HA1 yield compared with the yield of the 6:2 virus. When the outlier was discounted (as possibly resulting from an artefactually low recovery for the WT sample), average HA1 yield from the other eight experiments showed 1.9- and 2.4-fold improvements with the segment 3 FS mutation for Cal7 and Eng195, respectively (Table 1).

The HA yield of CVVs with pdm09 glycoproteins has been shown to be improved by engineering chimeric HA genes which contain signal peptide and transmembrane domain/cytoplasmic tail sequences from PR8 HA and the antigenic region of the HA gene from Cal7 (19, 20). To test if these gains were additive with those seen with the FS mutation, we introduced the NIBRG-119 construct, which is a segment 4 with the ectodomain coding region of Cal7 HA and all other sequences (3′ and 5′ noncoding regions, signal peptide, transmembrane domain, and cytoplasmic tail) from PR8 (19) into 6:2 CVV mimics with the WT A(H1N1)pdm09 NA gene and a PR8 backbone with or without the PA-X mutation. Viruses bearing the NIBRG-119 HA did not agglutinate chicken red blood cells (data not shown), so HA yield from eggs was assessed by SDS-PAGE and Western blotting of partially purified virus. Chimeric HA viruses containing the FS backbone showed an average HA yield improvement of 1.5-fold compared to the level of the WT backbone counterpart across independent small- and large-scale experiments (Table 1). Thus, the FS mutation is compatible with other rational strategies for increasing egg-grown reverse genetics vaccines.

Following on from this, several pairs of CVV mimics were made with glycoproteins from different IAV strains with either WT or FS mutant PR8 segment 3. These included viruses with glycoproteins of potentially pandemic strains, such as the highly pathogenic avian virus A/turkey/Turkey/1/2005 (H5N1) and low-pathogenic avian strains A/mallard/Netherlands/12/2000 (H7N3), A/chicken/Pakistan/UDL-01/2008 (H9N2), and A/mallard/Netherlands/10/99 (H1N1), as well as the human H3N2 strain A/Hong Kong/1/68 and an early seasonal H3N2 isolate, A/Udorn/307/72 (Table 1). HA yield in eggs was assessed from both the small-scale and large-scale experimental conditions described earlier by measuring HA titer and HA1 yield from partially purified virus particles. In general, the results of the two techniques were in agreement (Table 1). Ablating PA-X expression moderately improved HA1 yields of some of the CVVs tested: 1.5-fold for the avian H7N3 strain A/mallard/Netherlands/12/2000 and 1.3-fold for the human H3N2 A/Udorn/307/72 strain. Other CVVs showed smaller or effectively no increases. However, in no case did ablation of PA-X appear to be detrimental to the growth of CVVs.

## DISCUSSION

Here, we show that ablating expression of PA-X resulted in reduced pathogenicity in the chicken embryo model despite the PR8 PA-X protein having relatively low host cell shutoff activity compared to that of PA-X from other IAV strains. Although loss of PA-X expression had no effect on infectious titers in eggs, subtle differences in virion composition were observed, and, more importantly, the HA yield from poor-growing 6:2 reassortant vaccine analogues containing the HA and NA segments from A(H1N1)pdm09 strains was significantly improved.

The majority of studies examining the effect of loss of PA-X expression on IAV pathogenicity have used mice as the experimental system. As discussed above, in most cases, the outcome has been increased virulence (30, 34–40), but several studies have found the opposite effect, with PA-X deficiency reducing pathogenicity in mice (37, 41, 42). In adult bird challenge systems using chickens and ducks infected with a highly pathogenic H5N1 virus, abrogating PA-X expression caused increased virulence (35). In our infection model of embryonated hens’ eggs, loss of PA-X expression markedly reduced the pathogenicity in chick embryos. Thus, like PB1-F2, another *trans*-frame-encoded IAV accessory protein (52), the impact of PA-X expression on viral pathogenicity seems to vary according to both host and virus strains but not in a fashion that can simply be correlated with mammalian or avian settings.

In previous studies, changes in virulence phenotypes following loss of PA-X expression have been associated with its host cell shutoff function. In the virus strains used, whether from high-pathogenicity or low-pathogenicity IAV strains, the PA-X polypeptides were shown to significantly affect host cell gene expression. Here, despite PR8 PA-X failing to repress cellular gene expression, a strong phenotypic effect was seen in chicken embryos following loss of PA-X expression. Furthermore, these effects on pathogenicity were more pronounced in an otherwise WT PR8 virus than in a 7:1 reassortant with segment 3 from the highly pathogenic H5N1 avian influenza T/E strain, which encodes a PA-X with strong host cell shutoff activity. This lack of correlation between repression of cellular gene expression in avian cells and phenotypic effects in chicken embryos suggests that the PR8 PA-X protein may harbor a function unrelated to host cell shutoff. The PR8 PA-X protein has been proposed to inhibit stress granule formation but via a mechanism linked to its endonuclease activity and therefore presumably reflecting shutoff activity (53). Alternatively, it could be that the PR8 PA-X polypeptide exhibits a repressive function only in specific cell types, such as those of the chorioallantoic membrane (the primary site of virus replication in eggs) or the chicken embryo itself. However, since we found low shutoff activity from PA-X in a variety of cells from different species and, conversely, no great cell specificity for high-activity PA-X polypeptides (data not shown), we do not favor this hypothesis.

Several studies have found that sequences in the X ORF make positive contributions to the shutoff activity of PA-X (30, 37, 39, 46, 47). In contrast, here we found that for both PR8 and T/E strains of the polypeptide, removal of X ORF sequences actually increased shutoff activity compared to the level with the WT polypeptide. The effect was relatively modest and, in the case of PR8, did not confer equivalent activity to the full-length avian virus PA-X polypeptides (Fig. 2B). A similar outcome of greater inhibition from a truncated PA-X polypeptide was seen with a triple reassortant swine influenza virus (42), suggesting that the X ORF can harbor negative as well as positive regulatory polymorphisms.

In some but not all studies, effects of PA-X mutations on viral pathogenicity have been associated with differences in virus replication *in vivo*. While Jagger et al. (30) did not attribute the increased virulence in mice upon loss of 1918 H1N1 PA-X to virus replication, Gao and colleagues found that increased virulence in mice on loss of H5N1 PA-X was associated with increased titers of PA-X-deficient (ΔPA-X) viruses in the lungs, brains, and blood of infected mice (34, 39). Similarly, Hu et al. found that increased virulence in chicken, ducks, and mice of a ΔPA-X H5N1 virus was associated with increased virus titers in the host (35). Given the postulated role of PA-X-mediated repression of cellular gene expression in controlling host responses to infection, it is reasonable to hypothesize that these differing outcomes reflect the variable interplay between host and virus that is well known to tip in favor of one or the other depending on exact circumstance (54). Our present study, where loss of a PA-X protein with little apparent ability to modulate host gene expression had no significant effect on virus titers in allantoic fluid or the chick embryos themselves but nevertheless reduced pathogenicity, do not support this hypothesis. However, differences in progeny virion composition in the form of altered ratios of HA to NP and M1 between WT and FS viruses were seen. This may differentially affect their abilities to infect specific cell types as the amount of virus receptor varies between different tissue types and is a known determinant of tissue tropism of influenza viruses (reviewed in references 55 and 56).

Our findings have direct implications for HA yield of vaccine viruses in eggs. Ablating PA-X expression did not affect yield from eggs of high-growth viruses such as PR8 or from 6:2 reassortant CVV mimics containing glycoproteins of human H3N2 strains or from potentially pandemic low-pathogenicity avian H9N2 or H1N1 viruses. However, mutation of the PR8 PA-X gene in the background of a CVV analogue containing the HA and NA segments from poor-growing strains, such as A(H1N1)pdm09 viruses or a potentially pandemic avian H7N3 isolate, increased HA yield by around 2-fold. The mechanism of improved yield of certain virus subtypes but not others upon loss of PA-X expression is unclear. Other investigators have found that mutating the FS site of PR8 PA-X has subtle effects on viral protein expression *in vitro*, including lower levels of M1 (46), perhaps explaining the changes in the HA-to-M1 ratio we see. Beneficial outcomes to HA yield may be apparent only in low-yielding strains where perhaps viral rather than cellular factors are limiting. Alternatively, changes in virion composition between WT and FS viruses could result in subtype-/strain-specific effects depending on the balance between HA and NA activities (57). Whatever the mechanism, in no case was loss of PA-X expression detrimental to the yield of CVVs when the HA yield of a wide range of different influenza A subtypes/strains was assessed. This approach of modifying the PR8 donor backbone therefore potentially supplies a universal approach that can be applied to all CVVs that is additive with, but without the need for, generation and validation of subtype-/strain-specific constructs, as is required for strategies based on altering the glycoprotein genes. This could be beneficial to improve antigen yield in a pandemic setting where manufacturers are required to produce large amounts of vaccine quickly.

## MATERIALS AND METHODS

### Cell lines and plasmids.

Human embryonic kidney (293T) cells, Madin-Darby canine kidney epithelial cells (MDCK), and MDCK-SIAT1 (stably transfected with the cDNA of human 2,6-sialtransferase) cells (58) were obtained from the Crick Worldwide Influenza Centre, The Francis Crick Institute, London, United Kingdom. QT-35 (Japanese quail fibrosarcoma) cells (59) were obtained from Laurence Tiley, University of Cambridge. Cells were cultured in Dulbecco’s modified Eagle’s medium (DMEM) (Sigma) containing 10% (vol/vol) fetal bovine serum (FBS), 100 U/ml penicillin-streptomycin, and 100 U/ml GlutaMAX with 1 mg/ml Geneticin as a selection marker for the SIAT cells. Infection was carried out in serum-free DMEM containing 100 U/ml penicillin-streptomycin, 100 U/ml GlutaMAX, and 0.14% (wt/vol) bovine serum albumin (BSA). Cells were incubated at 37°C in 5% CO_2_. Reverse genetics plasmids were kindly provided by Ron Fouchier (A/Puerto Rico/8/34) (60), Wendy Barclay (A/England/195/2009 [61] and A/turkey/England/50-92/91 [62]), John McCauley (A/California/07/2009) (63), Laurence Tiley (A/mallard/Netherlands/10/1999) (64), Robert Lamb (A/Udorn/307/72) (65)), Earl Brown (A/Hong Kong/1/68) (66)), and Munir Iqbal (A/chicken/Pakistan/UDL-01/2008) (67). RG plasmids for A/mallard/Netherlands/12/2000 (NIBRG-60) and A/turkey/Turkey/1/2005 (NIBRG-23; with the multibasic cleavage site removed) (68) were made by amplifying HA and NA genes by PCR from cDNA clones available within the NIBSC and cloning them in a pHW2000 vector using BsmB1 restriction sites. A plasmid containing the *Renilla* luciferase gene behind the simian virus 40 early promoter (pRL) was supplied by Promega Ltd.

### Antibodies and sera.

Primary antibodies used were the following: rabbit polyclonal antibody anti-HA for swine H1 (Ab91641; Abcam), rabbit polyclonal anti-HA for H7N7 A/chicken/MD/MINHMA/2004 (IT-003-008; Immune Tech Ltd.), mouse monoclonal anti-HA for H5N1 (8D2 and Ab82455; Abcam), laboratory-made rabbit polyclonal anti-NP (2915) (69), anti-PA residues 16 to 213 (expressed as a fusion protein with β-galactosidase (70), anti-puromycin mouse monoclonal antibody (MABE343; Millipore), rabbit anti-PR8 PA-X peptide (residues 211 to 225) antibody (30), and anti-alpha-tubulin rat monoclonal antibody (MCA77G; Serotec). Secondary antibodies used were the following: for immunofluorescence, Alexa Fluor donkey anti-rabbit IgG 488 or 594 conjugates (Invitrogen); for immunohistochemistry, goat anti-mouse horseradish peroxidase (172-1011; Bio-Rad) and goat anti-rabbit horseradish peroxidase (172-1019; Bio-Rad); for Western blotting, donkey anti-rabbit IgG Dylight 800 or Alexa Fluor 680-conjugated donkey anti-mouse IgG (LiCor Biosciences).

### Site-directed mutagenesis.

A QuikChange Lightning site-directed mutagenesis kit (Stratagene) was used according to the manufacturer’s instructions. Primers used for site-directed mutagenesis of the segment 3 gene were designed using the primer design tool from Agilent technologies. The strategies used to disrupt the frameshift (FS) site as well as to generate C-terminally truncated versions of PA-X via PTCs were as described previously (30) (the cited study used the PTC1 construct).

### Protein analyses.

Coupled *in vitro* transcription-translation reactions were carried out in rabbit reticulocyte lysate supplemented with [^35^S]methionine using a Promega TNT system according to the manufacturer’s instructions. SDS-PAGE followed by autoradiography was performed according to standard procedures. Immunoprecipitations were performed as previously described (71). Transfection-based reporter assays to assess host cell shutoff by PA-X (described previously [30]) were performed by cotransfecting QT-35 cells with a reporter plasmid containing the *Renilla* luciferase gene along with pHW2000 plasmids expressing the appropriate segment 3 genes with or without the desired PA-X mutations. At 48 h posttransfection, cells were lysed, and luciferase activity was measured on a Promega GloMax 96-well microplate luminometer using the Promega *Renilla* luciferase system.

### Reverse genetics rescue of viruses.

All viruses used in this study were made by reverse genetics. 293T cells were transfected with eight pHW2000 plasmids, each encoding one of the influenza virus segments using Lipofectamine 2000 (Invitrogen). Cells were incubated for 6 h posttransfection before medium was replaced with DMEM serum-free virus growth medium. At 2 days posttransfection, 0.5 μg/ml tosylsulfonyl phenylalanyl chloromethyl ketone (TPCK)-trypsin (Sigma) was added to cells. Cell culture supernatants were harvested at 3 days posttransfection. 293T cell culture supernatants were clarified and used to infect 10- to 11 day-old embryonated hens’ eggs. At 3 days p.i., eggs were chilled overnight, and virus stocks were partially sequenced to confirm identity.

### RNA extraction, RT-PCR, and sequence analysis.

Viral RNA extractions were performed using a QIAamp viral RNA minikit with on-column DNase digestion (Qiagen). Reverse transcription used the influenza A virus Uni12 primer (AGCAAAAGCAGG) using a Verso cDNA kit (Thermo Scientific). PCRs were performed using Pfu Ultra II fusion 145 HS polymerase (Stratagene) or Taq polymerase (Invitrogen) according to the manufacturers’ protocols. PCR products were purified for sequencing by Illustra GFX PCR DNA and a Gel Band Purification kit (GE Healthcare). Primers and purified DNA were sent to GATC Biotech for Sanger sequencing (Lightrun method). Sequences were analyzed using DNAstar software.

### Virus titration.

Plaque assays, assays of the 50% tissue culture infective dose (TCID_50_), and hemagglutination assays were performed according to standard methods (72). MDCK or MDCK-SIAT cells were used for infectious virus titration, and infectious foci were visualized by either toluidine blue or immunostaining for viral NP and revealed using a tetramethyl benzidine (TMB) substrate.

### Virus purification and analysis.

Allantoic fluid was clarified by centrifugation twice at 6,500 × *g* for 10 min. Virus was then partially purified by ultracentrifugation at 128,000 × *g* for 1.5 h at 4°C through a 30% sucrose cushion. For further purification, virus pellets were resuspended in phosphate-buffered saline (PBS), loaded onto 15 to 60% sucrose-PBS density gradients, and centrifuged at 210,000 × *g* for 40 min at 4°C. Virus bands were extracted from gradients, and virus was pelleted by ultracentrifugation at 128,000 × *g* for 1.5 h at 4°C. Pellets were resuspended in PBS, and aliquots were treated with N-glycosidase F (New England Biolabs), according to the manufacturer’s protocol. Virus pellets were lysed in Laemmli sample buffer and separated by SDS-PAGE on 10% or 12% polyacrylamide gels under reducing conditions. Protein bands were visualized by Coomassie blue staining (Imperial protein stain; Thermo Scientific) or detected by immunostaining in Western blotting. Coomassie-stained gels were scanned, and bands were quantified using ImageJ software. Western blots were scanned on a LiCor Odyssey infrared imaging system, version 1.2, after they were stained with the appropriate antibodies, and bands were quantified using Image Studio Lite software (Odyssey).

### Chicken embryo pathogenesis model.

Ten-day old embryonated hens’ eggs were inoculated via the allantoic cavity route with 1,000 PFU in 100 μl per egg or were mock (serum-free medium only) infected. Embryo viability was subsequently determined by examination of veins lining the shell (which collapse on death) and embryo movement (for a few minutes). At 2 to 3 days p.i. (depending on experiment), embryos were killed by chilling, washed several times in PBS, and then scored blind for overt pathology by two observers in each experiment. Embryo scoring was as follows: 0, normal; 1, intact but with dispersed hemorrhages; 2, small, fragile embryo with dispersed hemorrhages. For histology, embryos were decapitated, washed several times in PBS, imaged, and fixed for several days in 4% formalin in PBS. Two embryos per virus condition were sectioned longitudinally and mounted onto paraffin wax. Tissue sections were cut and mounted onto slides and stained with hematoxylin and eosin (H&E) by the Easter Bush Pathology Service. Further sections were examined by immunohistofluorescence performed for influenza virus NP (63). Sections were deparaffinized and rehydrated, and heat-induced antigen retrieval was performed using sodium citrate buffer (10 mM sodium citrate, 0.05% Tween 20, pH 6.0). Sections were stained with anti-NP antibody followed by an Alexa Fluor-conjugated secondary antibody. Preimmune bleed serum was also used to confirm specificity of staining by anti-NP antibody. Sections were mounted using ProLong Gold antifade reagent containing 4′,6-diamidino-2-phenylindole (DAPI) (Invitrogen). Stained tissue sections were scanned using a NanoZoomer XR instrument (Hamamatsu) using bright-field or fluorescence settings. Images were analyzed using the NDP view, version 2.3, software (Hamamatsu).

### Graphs and statistical analyses.

All graphs were plotted, and statistical analyses (Mantel-Cox test, *t* tests, and Dunnett’s and Tukey’s tests as part of one-way analysis of variance [ANOVA]) were performed using GraphPad Prism software.
